# Fracture liaison service—a multidisciplinary approach to osteoporosis management

**DOI:** 10.1007/s00198-024-07181-7

**Published:** 2024-07-18

**Authors:** Hai V. Le, Benjamin W. Van, Hania Shahzad, Polly Teng, Nisha Punatar, Garima Agrawal, Bart Wise

**Affiliations:** 1 Department of Orthopaedic Surgery, University of California Davis School of Medicine, 4860 Y St #1700, Sacramento, CA 95817 USA; 2 Department of Endocrinology, University of California Davis School of Medicine, Sacramento, USA; 3 Department of Internal Medicine, University of California Davis School of Medicine, Sacramento, USA; 4 Department of Rheumatology, University of California Davis School of Medicine, Sacramento, USA

**Keywords:** Fragility fracture, Fracture liaison service, Fracture prevention, Osteoporosis, Osteoporotic fracture

## Abstract

A fracture liaison service is a systems-level multidisciplinary approach designed to reduce subsequent fracture risk in patients who recently sustained fragility fractures. It is estimated that one in three women and one in five men over the age of 50 years old have osteoporosis. Nonetheless, only 9 to 20% of patients who sustain an initial fragility fracture eventually receive any osteoporosis treatment. With the aim of preventing subsequent fractures, a fracture liaison service (FLS) works through identifying patients presenting with fragility fractures to the hospital and providing them with easier access to osteoporosis care through referrals for bone health and fracture risk assessment and recommendation or initiation of osteoporosis treatment. Currently, there are four major types of FLS models ranging from services that only identify at-risk patients and inform and educate the patient but take no further part in communicating their findings to other stakeholders in patients’ care, to services that identify, investigate, and initiate treatment at the other end of the spectrum. In this article, we review the benefits, challenges, and outcomes of FLS in the American healthcare system with further exploration of the roles each member of the multidisciplinary team can play in improving patients’ bone health.

## Introduction

Osteoporosis is a disease of progressive bone loss and skeletal deterioration in which bones become predisposed to fractures [1]. Worldwide, it is estimated that one in three women and one in five men > 50 years old have osteoporosis [2]. Annually, osteoporosis is the underlying pathology behind two million fractures. Additionally, the annual cost of treating osteoporosis-related fractures is the same or exceeds the cost of treating myocardial infarction, stroke, or breast cancer [3].

Fragility fractures (FFs) are defined as resulting from minimal trauma and often are the first indication that a patient has decreased bone mineral density (BMD). Ideally, patients would receive formal osteoporosis workup and management after sustaining an FF; however, rates of treatment after initial FF have been very poor, ranging from 9 to 20% [4]. Furthermore, up to 17.8% of patients who had a primary FF incurred a second FF within a median time of 555 days [5]. These statistics highlight the importance of secondary FF prevention as it can reduce patient morbidity and mortality as well as reduce healthcare burden. A fracture liaison service (FLS) attempts to achieve these secondary FF prevention outcomes through a systems-level multidisciplinary approach. This multidisciplinary approach involves collaboration among healthcare professionals from various specialties and allows for addressing the complex nature of osteoporosis and its management to allow for better patient outcomes, improved access to care, and increased efficiency in the healthcare system. In this paper, we review the benefits, challenges, and outcomes of FLSs in the American healthcare system.

## Osteoporosis diagnosis and management

According to the United States Preventive Screening Task Force, osteoporosis screening with BMD testing via dual-energy X-ray absorptiometry (DXA) scan is recommended in women > 65 years old [6]. Osteoporosis is defined by BMD at the lumbar spine, total hip, or femoral neck ≤ 2.5 standard deviations below BMD of a young-adult reference population (T-score ≤  − 2.5) [1]. The fracture risk assessment tool (FRAX) can be used to estimate the 10-year probability of a hip fracture or major osteoporotic fracture based on age, gender, medical history, and femoral neck BMD [7]. A FRAX estimate of hip fracture probability ≥ 3% or major osteoporotic fracture probability ≥ 20% warrants consideration of medical treatment [7]. Osteoporosis can be clinically diagnosed in patients who present with an FF with a recommendation for medical treatment to reduce the risk of subsequent fractures.

Osteoporosis treatment is tailored based on the severity of BMD loss and clinical manifestations of the disease. For patients with a normal BMD, no medical intervention is typically required; however, modifications such as avoiding smoking or excessive alcohol and maintaining a healthy lifestyle including adequate calcium and vitamin D intake and regular weight-bearing exercises are important for delaying or avoiding future fractures. Currently, the recommendation for calcium intake in women over age 51 is 1200 mg per day while in men ages 51–70 is 1000 mg per day, and in men older than 71 years is 1200 mg per day [8]. For vitamin D, it is recommended that all adults aged 51 to 70 years old receive 600 IU per day and adults over age 71 receive 800 IU per day [8].

Currently, available pharmacotherapies for osteoporosis include antiresorptive medications, such as bisphosphonates or denosumab, and agents with anabolic actions, such as teriparatide, abaloparatide, and romosozumab. Antiresorptive medications inhibit formation and function of osteoclasts while anabolic agents stimulate osteoblasts to promote bone formation. Several guidelines for osteoporosis treatment have been published with the consensus to initiate bisphosphonates for most osteoporotic patients with consideration of anabolic agents for those patients at very high fracture risk, such as those with severe or multiple vertebral fractures [9, 10].

## Rationale for secondary fracture prevention

Patients who do not sustain a second fracture will have reduced morbidity and mortality and, in turn, will have a better quality of life. Additionally, preventing a secondary fracture dramatically reduces the burden on the healthcare system. For Medicare beneficiaries, some secondary fracture prevention interventions would reduce the number of expected fractures by approximately 5% over a 5-year period, preventing 30,000 fractures for one million patients [11]. Furthermore, for one million patients who receive the intervention instead of usual care, the expected cost savings for Medicare would be 418 million dollars [11]. A proactive system such as an FLS is a model of care intended to effectively address the “osteoporosis care gap.”

## Current problems with osteoporosis management

There are several factors that can contribute to suboptimal inpatient osteoporosis management and secondary fracture prevention such as insufficient awareness of current osteoporosis guidelines, the belief that the efficacy of osteoporosis treatment has not yet been demonstrated, the health status of elderly patients who may have several comorbidities and be apprehensive about trying new treatments, and perception among providers that osteoporosis treatment is not their responsibility, perhaps compounded by a shortage of physician time [12–14]. While continued education of physicians and patients can help address some of the factors mentioned above, it is crucial to recognize that the osteoporosis care gap extends beyond educational gaps. Systemic challenges such as institutional siloing, limited availability of DXA scans, and the lack of access to specialists for overseeing complex osteoporosis care also contribute to this gap [15].

Osteoporosis treatment is the responsibility of all members of an FF patient’s care; however, additional help such as an inpatient osteoporosis consultation team may be beneficial. In one study, osteoporosis consultations helped facilitate the recognition of secondary causes and treatment of osteoporosis. The majority of patients who were started on treatment were found to continue the medication after discharge [16].

There are several interventions that can be used by the inpatient care team for patients with FFs. First, initiation of calcium and vitamin D should be the standard of care to ensure adequate stores and reduce the risk of secondary hyperparathyroidism and osteomalacia. Second, ensuring adequate nutritional status is important, especially in the elderly population. Other items to consider are addressing a patient’s risk factors for falling and doing a medication reconciliation to identify and potentially discontinue medications that may increase fall risk. The initiation of pharmacologic treatment for osteoporosis should be considered in the inpatient setting after a FF as it often does not need to be delayed until the fracture heals. Bisphosphonates are the most commonly used osteoporosis medications though anabolic agents such as teriparatide and monoclonal antibodies such as denosumab are commonly utilized as well.

## Osteoporosis care gap and barriers

The osteoporosis care gap refers to the discrepancy between the number of patients who have osteoporosis and the number of patients who receive treatment for osteoporosis. For example, Haffner et al. showed that only 19% of patients ≥ 60 years old with vertebral compression fractures after low-energy traumas were initiated on pharmacologic therapy [17]. Currently, screening and treatments for osteoporosis are often underutilized due to barriers such as lack of access to care, confusion about reimbursement policies, fragmented care, and lack of data on racial and ethnic differences in osteoporosis risk and treatment responses [18]. Additionally, other reasons why this care gap exists are due to the cost of diagnosing and treating osteoporosis, the risks and concerns of polypharmacy, and the lack of clarity about which specialty manages these patients. When barriers were explored further, the reason why 48.2% of patients did not receive osteoporosis medications after 1 year of FF workup was because their healthcare providers neither discussed nor initiated treatment [19]. Thus, an emphasis on education for providers is warranted.

A recent white paper published by the National Committee for Quality Assurance reviews solutions to overcome such barriers and can be summarized as [18]:

1. Access to screening and treatment—Improving access to DXA scans through grants and leveraging telemedicine for practices in rural communities.
2. Provider support—Educating providers about osteoporosis-related topics such as racial and ethnic differences, overcoming ageism biases, and how to obtain preauthorization.
3. Shared decision-making—Providing regular visits to establish relationships, assess treatment tolerance and adherence, and change medications when needed.
4. Multidisciplinary team-based care—Involving other members of the healthcare system such as pharmacists, geriatricians, and physical therapists for osteoporosis management after a fracture.
5. Incentives—Using performance and quality metrics linked to reimbursement to ensure accountability in quality care.

## Function of a fracture liaison service

**Fig. 1:**
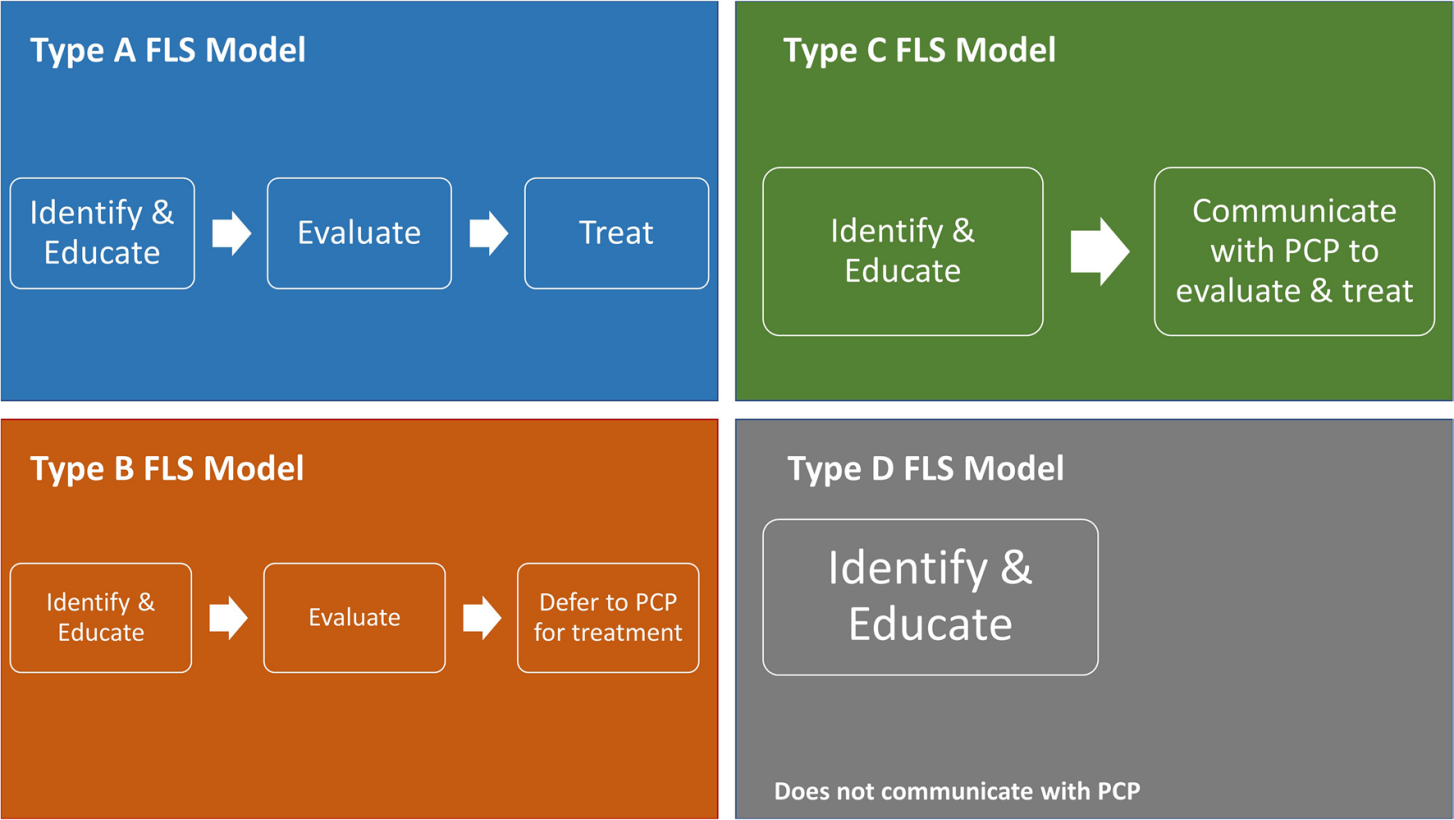
The 4 FLS models as described by Ganda et al. [22]

FLS was first established in the UK in the late 1990s to address the osteoporosis care gap [20]. By following patients who sustain FFs from the time of injury until care is transitioned over to the primary care provider (PCP), an FLS ensures that patients with clinical signs of osteoporosis receive appropriate evaluation and treatment [21]. Upon initial presentation to the hospital after an FFe, patients will get worked up by the emergency medicine physician and usually admitted into a hospitalist medicine service or an orthopedic co-management service. The orthopedic surgeon will surgically stabilize the fracture and soon afterwards, FLS care will be initiated. Throughout the patient’s hospital course, an FLS coordinator will meet with the patient and begin the process of osteoporosis education, evaluation, and management. Consulting services such as endocrinology and rheumatology may provide additional recommendations depending on the underlying cause of the patient’s osteoporosis. Additional services such as physical therapy, nutrition, and pharmacy may also evaluate the patient on fall risk, nutritional deficiencies, and polypharmacy or optimal medication regimen. Supplements such as calcium and vitamin D are usually started during the inpatient course as well. After the patient is discharged from the hospital, the FLS coordinator continues to manage the patient’s post-operative care among other stakeholders to obtain DXA imaging and/or further management such as additional medication, continued physical therapy, or nutritional evaluations. Once the patient is deemed healthy enough to be discharged from FLS care, one major responsibility that the FLS coordinator has is to help transition care back to the PCP for long-term osteoporosis management. Currently, there are four types of FLS models as described by Ganda et al. (Fig. 1) [22].

- Type A model is an FLS that identifies, investigates, and initiates treatment.
- Type B model is an FLS that identifies and investigates patients but then refers back to the PCP for treatment initiation.
- Type C model is an FLS that identifies patients at risk and informs them and their PCP. However, Type C FLS programs do not undertake any assessment or treatment of the patients.
- Type D model is an FLS that identifies at-risk patients and informs and educates them but takes no further part in communicating their findings to other stakeholders in the patient’s care.

When comparing the types of FLS models, medical centers that employ more intensive services in which they take full responsibility for investigation and treatment achieve better results than less intensive services. Specifically, the more intensive FLS models have been found to reduce the re-fracture risk, reduce mortality, increase BMD assessment, increase treatment initiation, and increase treatment adherence [22].

## Key stakeholders in a fracture liaison service

There are several key stakeholders in a FLS and each one has a unique role to play in the success of the FLS (Table 1).

1. Patients: FLSs are focused on patients with FFs; thus, they are the most important stakeholders. The role of the patient is to actively participate in the evaluation and management of their bone health to prevent worsening osteoporosis or the development of a secondary FF. The biggest challenge that patients, especially geriatric patients, face is keeping track of the multidisciplinary care they are receiving; therefore, additional help through assigned nurse case managers can help.
2. Emergency medicine: Emergency medicine physicians are the first points of contact when a patient arrives at the hospital after a FF. When these patients arrive, emergency medicine physicians stabilize the patient and are the first ones to order diagnostic imaging of the fracture. If prepared to do so within their system of care, emergency medicine physicians can clinically diagnose osteoporosis and consult other members of the FLS team for further workup of bone health.
3. Hospital medicine: Internal medicine hospitalists, like emergency medicine physicians who screen for patients with osteoporosis, are frequently engaged in co-managing surgical patients and can take on the role of coordinating post-fracture osteoporosis care. Results of a recent hospitalist-led FLS drastically improved quality metrics for elderly patients with osteoporotic hip fractures. In this study, Drabkin et al. reported 74% of those under the FLS care vs 11% of eligible patients (based on adequate renal function and vitamin D stores) without FLS care were discharged with anti-osteoporosis medications (*p* < 0.001), 82% vs 38% were discharged with vitamin D/calcium supplements (*p* < 0.001), 22% vs 5% underwent a DXA scan after discharge (*p* < 0.05), and 65% vs 0% were referred to outpatient osteoporosis-specific care at discharge (*p* < 0.001) [23].
4. Orthopedic surgery: Orthopedic surgeons can play many key roles in the FLS team. First, in the inpatient setting, orthopedic surgeons surgically stabilize the FFs so patients can regain function. Additionally, since orthopedic surgeons can identify FF patterns and define which ones are most likely due to decreased BMD, they can screen for osteoporosis and identify patients who are at risk for future fractures. Traditionally, once the patient is discharged, they will follow up with their orthopedic surgeon in the outpatient setting for up to a year. During the post-discharge period would be an optimal time for the orthopedic surgeon to initiate bone health workup and anti-osteoporosis medications though there has been some ambiguity in this area due to the finite nature of this relationship and some authors suggest that other specialties should take over the medical management [21, 24]. Regardless, orthopedic surgeons should discuss bone health with this patient population when they are able to and consider starting patients on osteoporosis medications if not contraindicated.
5. Primary care physician (PCP): Primary care physicians are central to the FLS as they collaborate with specialists to refer patients for BMD testing and initiate osteoporosis plans. Additionally, they are vital in longitudinal care for patients as they monitor patients’ progress, manage comorbidities, and promote treatment adherence to reduce the risk of future fractures.
6. Geriatrician: Geriatricians provide specialized care for older adults who have sustained fragility fractures with emphasis on considering factors such as frailty, functional status, and polypharmacy. They are able to tailor treatment plans to their patient’s unique needs, focusing on medication tolerability and functional fall prevention strategies. Through collaborating with the FLS team, geriatricians optimize osteoporosis management and enhance the quality of life for patients.
7. Endocrinology and rheumatology: Endocrine disorders such as diabetes mellitus, hyperparathyroidism, and hyperthyroidism constitute the most frequent cause of secondary osteoporosis in men and women [25]. Numerous rheumatic diseases, including rheumatoid arthritis, psoriatic arthritis, ankylosing spondylitis, systemic lupus erythematosus, systemic sclerosis, dermatomyositis/polymyositis, and vasculitis, are characterized by osteoporosis and FFs as well [26]. In the inpatient setting, endocrinologists and rheumatologists can serve as consulting services to the FLS. These specialists can evaluate for the presence of secondary osteoporosis and provide individualized recommendations for osteoporosis treatment. In the outpatient setting, patients with osteoporosis can then establish care with these physicians for continued longitudinal monitoring and follow-up for the underlying disease and the decreased BMD.
8. Pharmacy: There are two main roles that pharmacists play in the FLS. Their first responsibility is to ensure that patients are receiving appropriate medications to treat their osteoporosis and reduce the risk of future fractures. Additionally, they can perform thorough medication reconciliations to see if there are any medication interactions or side effects, as most patients who sustain an FF are geriatric and have multiple medical comorbidities. Pharmacists should also optimize medication regimens using the American Geriatric Society (AGS) Beers Criteria so that patients have decreased chances of experiencing a ground-level fall as this is the most common mechanism behind an FF [27].
9. Physical therapy: The role of a physical therapist in an FLS is twofold. First, in the immediate post-operative period, they help patients achieve early mobility to reduce post-operative complications such as thromboembolism, pneumonia, wound breakdown, pressure ulcers, and delirium [28]. After the immediate post-operative period, the role of a physical therapist shifts towards regaining strength and improving BMD through personalized exercise plans. Regaining strength, especially in the lower extremities, will help locomotion stability and may help prevent another fall. Additionally, physical therapists can educate patients on fall prevention strategies and provide assistive devices to help patients maintain mobility and independence.
10. Nutrition: The role of a nutritionist in an FLS is to provide education and guidance on proper nutrition to improve bone health. Nutritionists can create individualized nutrition plans with patients that emphasize obtaining enough calcium and vitamin D through dietary means and can provide recommendations on supplemental vitamins. Additionally, nutritionists educate patients about the impact of alcohol on bone health, as excessive alcohol consumption can impair bone healing and increase the risk of fractures. Moreover, it is important to educate patients that alcohol intoxication contributes to the likelihood of falls, which can further increase the risk of FFs. Furthermore, nutritionists can work with patients on weight management and reduction strategies, which play a crucial role in preventing future FFs.
11. Institution: The institution hosting the FLS supports the delivery of care through providing infrastructure and resources such as clinic space and diagnostic equipment. Additionally, it fosters interdisciplinary collaboration among healthcare professionals and supports ongoing education and training initiatives. Through quality improvement initiatives, the institution can continuously improve the delivery of care and outcomes for patients with osteoporosis.

**Table 1:** Roles of key stakeholders in a fracture liaison service

Stakeholder	Role in evaluation and management of care
Patients	Actively participate in the evaluation and management of their bone health, follow recommendations by healthcare professionals, and track their care
Emergency medicine	Stabilize the patient, order diagnostic imaging, clinically diagnose osteoporosis, and consult other FLS team members
Hospital medicine	Co-manage surgical teams, screen for osteoporosis, coordinate post-fracture osteoporosis care, and improve quality metrics
Orthopedic surgery	Surgically stabilize fractures, screen for osteoporosis, initiate bone health workup, and consider osteoporosis medications
Primary care physician (PCP)	Provide longitudinal care to monitor patient progress, manage comorbidities, and promote treatment adherence
Geriatrician	Tailor treatment plans to patients’ unique needs focusing on medication tolerability and optimization as well as enhancing quality of life
Endocrinology and rheumatology	Evaluate for secondary osteoporosis, provide individualized treatment recommendations, and establish long-term care
Pharmacy	Ensure appropriate medications, perform medication reconciliation, optimize medication regimens, and reduce medication risks
Physical therapy	Aid in early mobility post-surgery, improve strength and bone mineral density through exercise, and education on fall prevention
Nutrition	Provide nutrition education and guidance, create individualized nutrition plans, recommend supplements, and assist in weight management
Institution	Provide infrastructure and resources as well as foster interdisciplinary collaborating among healthcare professionals to support ongoing education and training initiatives

## Challenges of a fracture liaison service

There are several challenges that a FLS may encounter:

First, a “bone health champion” needs to be identified and recruited. This bone health champion is someone who can initiate the FLS, manage an identification system to find FF patients and coordinate outpatient services. This position could be filled by a physician or midlevel provider and ideally would be able to order labs and BMD testing and prescribe osteoporosis medications. Additionally, for an FLS to be successful, there needs to be a constant input of effort for osteoporosis education and reinforcement of the importance of the program. Settings that have high turnover such as teaching hospitals with residents and fellows or settings with clinicians who are not interested in osteoporosis or who are not interested in what happens after surgical repair and hospital discharge are unlikely to be successful in establishing an effective and lasting FLS service. To help alleviate some of this problem, the International Osteoporosis Foundation (IOF) has created the “Capture the Fracture” campaign providing best practice frameworks on ways to establish an FLS [29].

The next challenge is identifying patients who would benefit from the FLS in multiple settings such as an orthopedic service, a medical service where orthopedic surgeons are consultants and emergency departments. Each setting will require “buy-in” from all relevant stakeholders in identifying patients in real time with osteoporosis and initiating the FLS. Moreover, regardless of the setting, the FLS will greatly benefit from the support of a collaborative information technology (IT) department in generating weekly lists of patients with fragility fractures based on ICD-10 codes. However, relying solely on ICD-10 coding for identifying FFs has its limitations, as they are often not accurately coded. Simultaneously employing artificial intelligence (AI) within radiology software or utilizing SNOMED codes to analyze admission diagnoses and radiology reports will enhance the identification of patients with FFs. These approaches are currently undergoing further development.

Another challenge in successfully implementing an FLS can be covering the salary of an FLS provider. In the United States, most healthcare systems receive a single payment for fracture repair and this bundled payment must encompass all services and disincentivizes “extra” care that is not directly related to fracture fixation; thus, osteoporosis management is not typically reimbursed in the inpatient setting. Some of this can be overcome by using coding modifiers that separate osteoporosis evaluation and management from the global surgery payment. Additionally, cost savings from a reduction in the number of secondary fractures and incremental increases in office visits as well as ancillary income from laboratory, radiology, and pharmacy services can help yield cost neutrality in an FLS program [30].

Several FLSs have conducted formal cost analyses. One example is the Healthy Bones Program run by the Kaiser Southern California health-maintenance organization (HMO). This program in 2006 showed a 37.2% reduction in hip fracture rates in their regional HMO which equals 935 hip fractures prevented. Given that the average cost of treating a hip fracture is $33,000, the organization was able to save more than $30.8 million during the 2006 fiscal year [31].

Lastly, partnership with patients’ PCPs is crucial in the longitudinal management of osteoporosis care. PCPs sometimes do not understand the purpose of an FLS or feel that it is intrusive and may withhold full personal participation. Additionally, if PCPs view osteoporosis or FFs as inevitable comorbidities of old age, longitudinal care of patients’ bone health will be compromised. Therefore, another responsibility of an FLS is to educate other providers, especially during transitions of care from inpatient to outpatient settings. A recent systematic literature review demonstrated that a successful FLS has several components with the most important being multidisciplinary involvement, driven by a dedicated case manager, regular assessment and follow-up, multifaceted interventions, and patient education [32].

## Fracture liaison services in the United States

The specific number of FLSs in the United States is not readily available as it can vary over time and is not consistently tracked by a centralized authority. However, data from 2018 indicates that there were 240 established FLS sites in the United States through the American Orthopaedic Association (AOA) “Own the Bone” campaign. Several regional-based FLSs have shown promising results [31, 34–36]. Additionally, previous meta-analyses about FLSs have highlighted challenges in interpreting outcome measures from FLS due to reporting heterogeneity [37]; therefore, the “Own the Bone” campaign also aims to establish a comprehensive FLS registry to standardize processes and outcome measures.

## Outcomes of a fracture liaison service

### Future fracture risk reduction

When comparing an FLS to either PCP follow-up or a comparable hospital without an FLS program, studies have reported significant reductions in subsequent fractures over 2–4 years following the initial fracture with a recent meta-analysis evaluating eight papers revealing that FLS care was associated with a 30% lower probability of subsequent fractures [38]. Another meta-analysis reported that the unweighted average rates of re-fracture were 13.4% in the control arm and 6.4% in the FLS arm [33]. In the United States, one notable program is the Kaiser Permanente Southern California Healthy Bones Program, an FLS service that identifies, investigates, and initiates outpatient osteoporosis treatment. Outcomes published from this program show an average reduction of re-fracture rate of 37.2% over the first 4 years collected from 11 medical centers within their system [34]. Subsequent analyses show a 38.1% reduction in expected hip fractures [35]. FLS models that are less intense and focus on improving patient and physician knowledge of bone health unfortunately have not demonstrated any improvement in re-fracture rates [22].

### Bone health assessment

When compared to usual care, an FLS is associated with a 2- to 18-fold increase in the number of patients referred for bone density assessment with DXA [22] and was found through meta-analyses to have a risk difference of 43% (95% CI 23–64%) [38]. One study based out of Columbia, New York, found that initiation of an FLS during hip fracture rehabilitation increased BMD testing from 35 to 65% [35]. The Kaiser Permanente Southern California FLS reported a 246% increase in total annual DXA scans over the first 4 years and a 263% increase in total annual DXA scans over the first 6 years [34]. Additionally, a systematic review and meta-analysis reported that when compared with patients receiving usual care, patients receiving care from an FLS program had higher rates of BMD testing (48.0% vs 23.5%) [33]. Similar to future fracture risk reduction, FLS models that are less intensive such as only employing educational programs have not shown robust results; therefore, being able to initiate bone health assessment as part of an inpatient FLS is paramount for BMD assessment being completed [39].

### Osteoporosis treatment initiation and adherence

Outcomes regarding osteoporosis treatment used in studies have primarily been the rates of initiation of pharmacotherapy and rates of pharmacotherapy adherence at later points in time. Currently, though there is some heterogeneity in the findings, orthogeriatric care was associated with higher odds of initiation of calcium and vitamin D supplements and discharge on anti-osteoporosis medication [40]. Additionally, meta-analyses demonstrated significant efficacy for interventions of an FLS (risk difference 20%, 95% CI 1–40%) [41]. An FLS program in Edmonton, Canada, demonstrated increased rates of post-hip fracture bisphosphonate prescriptions from 22% in control patients who only received education to 51% after 6 months following the implementation of a case manager who counseled patients about the importance of BMD testing and the benefits of pharmacologic therapy [42]. Additionally, when evaluating wrist fractures, the same FLS showed an increase in bisphosphonate prescription rate from 7% in control patients who only received education to 22% after 6 months following a multifaceted approach of counseling patients and mailing reminders and osteoporosis guideline letters to PCPs [43].

Even when prescriptions were not written by the FLS but treatment recommendations were made by the FLS to the PCP, Axelsson and colleagues reported after 1 year an increased rate of medical treatment from 12.6% in control patients who only received education to 31.8% in patients who went through their four-step FLS consisting of patient identification, BMD assessment, BMD evaluation, and treatment initiation [44]. In contrast, when no treatment recommendations were made by less intensive FLS models and only educational programs were done, there was no difference in BMD testing rates or osteoporosis medication prescription rates when comparing the educational program group to the routine post-fracture care control group [39].

While the initiation of medical osteoporosis treatment is important, long-term adherence to the treatment plan is vital for the prevention of subsequent fractures. An FLS allows for a longitudinal care team solely dedicated to a patient’s bone health; therefore, it is expected that treatment adherence rates be greater in patients with follow-up in an FLS compared to patients who follow up with their PCP; however, a meta-analysis of 60 studies that reviewed adherence to oral bisphosphonates revealed that at the 1-year after medication initiation, adherence rates ranged between 17.65 and 74.80% while several other studies looking at 1-year adherence rates for osteoporosis medications within an FLS range between 44 and 80% [4, 45]. Further studies should aim to evaluate the differences in medication adherence between an FLS cohort and a non-FLS cohort to see if there are any areas for improvement that other FLSs can implement in their practice.

## Summary

In conclusion, an FLS is a multidisciplinary approach to osteoporosis management aimed at reducing subsequent fracture risk and increasing rates of bone health assessment and osteoporosis treatment initiation and adherence. This approach involves the identification of patients with FFs and providing them with easier access to comprehensive osteoporosis care. The involvement of many key stakeholders is needed for the success of an FLS. While the benefits of an FLS are significant, challenges still exist including finding a primary FLS provider, recruiting patients who sustain an FF, and finding funding to sustain the program over time. This multidisciplinary approach to osteoporosis care is crucial in closing the osteoporosis care gap; therefore, additional efforts should be made to further promote and improve it.
